# Phenotypic and Genotypic Characterization of *Escherichia coli* and *Salmonella* spp. Isolates from Pigs at Slaughterhouse and from Commercial Pork Meat in Portugal

**DOI:** 10.3390/antibiotics13100957

**Published:** 2024-10-11

**Authors:** Carlota Gonçalves, Leonor Silveira, João Rodrigues, Rosália Furtado, Sónia Ramos, Alexandra Nunes, Ângela Pista

**Affiliations:** 1 National Reference Laboratory for Gastrointestinal Infections, Department of Infectious Diseases, National Institute of Health Doutor Ricardo Jorge, Avenida Padre Cruz, 1649-016 Lisbon, Portugal; carlotamateusgoncalves@outlook.pt; 2 Department of Chemistry, Nova School of Science & Technology, Campus da Caparica, 2829-516 Caparica, Portugal; 3 Laboratory of Microbiology, Department of Infectious Diseases, National Institute of Health Doutor Ricardo Jorge, Avenida Padre Cruz, 1649-016 Lisbon, Portugal; joao.rodrigues@insa.min-saude.pt; 4 Food Microbiology Laboratory, Food and Nutrition Department, National Institute of Health Doutor Ricardo Jorge, Avenida Padre Cruz, 1649-016 Lisbon, Portugal; rosalia.furtado@insa.min-saude.pt; 5 Animal and Veterinary Research Centre (CECAV), Faculty of Veterinary Medicine, Lusófona University—Lisbon University Centre, 1749-024 Lisbon, Portugal; p2412@ulusofona.pt; 6 Genomics and Bioinformatics Unit, Department of Infectious Diseases, National Institute of Health Doutor Ricardo Jorge, Avenida Padre Cruz, 1649-016 Lisbon, Portugal

**Keywords:** *E. coli*, *Salmonella*, antimicrobial resistance, pork, food safety, WGS

## Abstract

**Background:** Foodborne diseases are a serious public health concern, and food-producing animals are a major source of contamination. **Methods:** The present study analysed *Escherichia coli* and *Salmonella* spp. isolated from faecal samples of 100 fattening pigs and from 52 samples of pork meat. **Results:** The results showed that the majority of the analysed meat samples were considered satisfactory in terms of microbiological quality (92.3% for *E. coli* and 94.2% for *Salmonella* spp.). *Salmonella* spp. was identified in 5.8% of the meat samples, whereas *E. coli* was detected in 89.5% of all samples (69.2% in meat and 100% in faecal samples). Furthermore, 1.9% of the faecal samples contained Shiga-toxin-producing *E. coli* and 3.9% contained enterotoxigenic *E. coli*. All sequenced isolates presented virulence genes for extraintestinal pathogenic *E. coli*. Moreover, 75.0% of *E. coli* isolates from meat and 71.8% from faeces samples showed antibiotic resistance, with 40.7% and 51.4%, respectively, being multidrug-resistant (MDR). The most prevalent resistances were to tetracycline, ampicillin, and sulfamethoxazole, and one *E. coli* isolate showed resistance to extended-spectrum β-lactamase. **Conclusions:** This study highlights the role of pigs as a potential source of human contamination and the importance of a One Health approach to ensure food safety and to promote public health.

## 1. Introduction

Food safety is essential for public health promotion and protection, and relies on preventive measures and food safety regulations to ensure the safety of food and, consequently, the health of consumers [1,2,3,4]. Food- and waterborne diseases are caused by the ingestion of food or water contaminated with infectious agents or with toxins produced by these agents [3]. According to the World Health Organization (WHO) and the Food and Agriculture Organization of the United Nations (FAO), each year, 600 million cases are foodborne-associated, more than 420,000 people die as a result of food- and waterborne diseases, and 125,000 of those deaths are of children under 5 years old [5].

*Salmonella* spp. and *Escherichia coli*, specifically Shiga-toxin-producing *E. coli* (STEC), are two of the main causes of food- and waterborne diseases and have been responsible for several outbreaks around the world [6]. Pork accounts for 33% of the meat consumed globally, and the swine industry carries significant economic weight in Portugal [7,8,9]. However, pork is a major source of transmission of pathogens, including *Salmonella* spp. and *E. coli* [3,10]. According to the European Food Safety Authority and the European Centre for Disease Prevention and Control (EFSA/ECDC) 2022 zoonoses report, 24 outbreaks of *Salmonella* spp. were strongly associated with the consumption of pork meat and the products thereof, while, for *E. coli*, 2.0% of the fresh pork meat tested positive for STEC [6]. The contamination of such foodstuffs can occur at any stage of the food chain, including primary production, processing, distribution, and preparation, but the environment in slaughterhouses has been identified as the most favourable stage for it to occur [7,11,12,13,14,15,16,17].

In recent years, there has been a significant increase in antimicrobial-resistant microorganisms, and antimicrobial resistance (AMR) is now considered one of the main public health problems of the 21st century. The WHO has ranked AMR as one of the top 10 global public health threats [18,19], with a particular concern regarding the role of multidrug-resistant (MDR) bacteria, driven by plasmids and mobile genetic elements that can be transferred within a bacterial species or sometimes across the interspecies barrier. In Europe, approximately 35,000 people die each year from untreatable infections caused by MDR bacteria [20], and it is estimated that, without intervention, by 2050, 10 million deaths *per* year will occur, mostly associated with Gram-negative MDR bacteria [21]. Antimicrobial resistance occurs naturally but the excessive and inappropriate use of antimicrobials in veterinary clinical practice, most specifically in food-producing animals, plays a significant role in the dissemination of resistance markers in ecosystems [20,22]. Indeed, antimicrobials administered in animal production are excreted into the environment, where they can exert selective pressure that increases antimicrobial resistance [23,24]. Several studies have identified extended-spectrum β-lactamase (ESBL)-producing *E. coli* and MDR *Salmonella* spp. in swine and pork meat [11,25,26]. In Portugal, antibiotic sales for food-producing animals decreased from 159.9 tons in 2021 to 82 tons in 2022, with tetracyclines, penicillins, and macrolides being the most sold antibiotic classes [27].

The objective of this study was to broaden our knowledge about the presence of *E. coli* and *Salmonella* spp. in pig faeces from slaughterhouses and pork meat acquired from retail stores in Portugal, generating additional data that may contribute to strengthening the surveillance of these zoonotic agents. Our goal was also to perform a genomic characterisation of the isolates using Whole-Genome Sequencing (WGS), to gain insight into the diversity of pathotypes, phylogroups, serotypes, and plasmid replicons, as well as the distribution of the virulence and antimicrobial resistant genes.

## 2. Results

In the present study, a total of 152 samples (100 pig faeces and 52 raw pork meat) were independently collected between November 2022 and April 2023. The samples were evaluated for the presence and characterisation of pathogenic *E. coli* and *Salmonella* spp.

### 2.1. Microbiological Quality of Pork Meat

The study analysed 52 food samples for the *E. coli* count (cfu/g) and the presence of *Salmonella* spp. These samples were classified as satisfactory, borderline, or unsatisfactory according to their microbiological quality, as defined in Regulation (EC) 1441/2007 [28]. Most of the analysed meat samples were considered satisfactory in terms of microbiological quality, 92.3% for *E. coli* and 94.2% for *Salmonella* spp. (Table 1). It was noted that the meat samples which tested positive for *Salmonella* spp. also contained *E. coli*, but at levels considered microbiologically satisfactory.

### 2.2. E. coli and Salmonella spp. Isolation and Typing

Overall, *E. coli* was isolated in 89.5% (136/152) of the samples and *Salmonella* spp. in 2.0% (3/152). Specifically, *E. coli* was recovered from 69.2% (36/52) pork meat samples (including samples with less than 10 cfu/g) and *Salmonella* spp. from 5.8% (3/52). However, while all pig faeces samples presented *E. coli* (100%), no *Salmonella* isolates were identified (Table 2).

Regarding the pathogenicity of *E. coli*, 40.1% (61/152) of the samples presented intestinal pathogenic *E. coli* (IPEC). Most specifically, STEC and enterotoxigenic *E. coli* (ETEC) were identified in 1.3% and 2.6% of the samples, respectively, all recovered from faeces. No IPEC was detected in pork meat. Additionally, 36.2% (55/152) of the samples presented isolates classified as extraintestinal pathogenic *E. coli* (ExPEC), mostly in faeces (41.0% vs. 26.9% in meat) (Table 2; Supplementary Table S1).

In faecal samples, WGS allowed the identification of the subtype *stx2e*, while the gene *eae* was not found in either isolate (Supplementary Table S1). It also allowed the identification of the *estb-STb1* gene (ETEC) in four samples (Supplementary Table S1). Fifty-five isolates (36.2%) were classified as ExPEC, including the six IPEC isolates already described (Table 2, Supplementary Table S1). Notably, three samples contained different pathogenic *E. coli* isolates each: in sample SM164, one isolate was identified as STEC (Ec_SM164-1) and the other as ExPEC (Ec_SM164-3), while samples SM8 and SM43 contained both ETEC (Ec_SM8-1 and Ec_SM43-5) and ExPEC (Ec_SM8-3 and Ec_SM43-2) isolates (Supplementary Table S1).

In the case of *E. coli*, WGS revealed a high diversity of sequence types (STs), with 13 STs found in meat and 30 STs in faeces. The most prevalent ST in pork meat was ST1115 (14.3%; 2/14), while, in isolates from faeces, it was ST101 (17.0%; 8/47). Interestingly, ST10, 88, and 101 were identified in both sample types. Moreover, three novel STs (ST4764, ST14765, and ST14766) were identified in *E. coli* from meat (Supplementary Table S1). Thirteen serotypes were identified in meat and 32 in faeces, with O102:H40 (14.3%; 2/14) and O8:H9 (8.5%; 4/47) being the most prevalent, respectively (Supplementary Table S1). Regarding the IPEC strains, three STs and four serotypes were identified in ETEC isolates (ST101/O1:H8; ST763/O157:H19; ST641/O45:H10; and ST641/O137:H10). Additionally, two STEC isolates shared the same ST (ST88) and the same serogroup (O8) but were assigned to different serotypes: O8:H9 and O8:H19. Unfortunately, the serotype could not be identified (ONT—O antigen non-typable) in eight *E. coli* isolates recovered from faeces (17.0%; 8/47).

Most of the *E. coli* isolates were classified as belonging to phylogroups B1 (44.3%; 27/61) and A (37.7%; 23/61). Specifically, in isolates from meat samples, phylogroup A accounted for 42.9% and phylogroup B1 accounted for 21.4%, while, in isolates from faeces, phylogroup B1 accounted for 51.1% and phylogroup A accounted for 36.2% (Supplementary Table S1). The remaining isolates were divided into groups C (6.6%), E (3.3%), G (3.3%), B2 (1.6%), D (1.6%), and F (1.6%). All the ETEC isolates were associated with phylogroup B1 (4/4), and the STEC isolates with phylogroup C (2/2).

The three *Salmonella* isolates identified in pork meat were serotyped as *S.* London (ST155), *S.* Typhimurium (ST19), and a monophasic variant of *S.* Typhimurium (ST34) (Supplementary Table S1). Interestingly, the co-occurrence of ExPEC and *Salmonella* (*S.* London and *S.* monophasic Typhimurium) isolates were detected in two meat samples (CC10 and CC47).

### 2.3. Virulence Determinants

The *E. coli* isolates that were studied were found to harbour multiple virulence genes. In total, 74 different virulence genes were identified in the *E. coli* isolates (Supplementary Table S1). In addition to the virulence genes associated with STEC (*stx2e*; 2.0%) and ETEC (*estb-STb1*; 4.0%), several other genes associated with adhesins, fimbriae, protectins, toxins, and siderophores, which are characteristic of ExPEC pathotypes, were also identified. Some genes such as *capU*, *cnf1*, *colE9*, *dhak*, *espP*, *hlyA*, *ibeA*, *kpsE*, *kpsMII*, *usp*, and *yfcV* were exclusively found in *E. coli* isolates from meat. On the other hand, genes like *aaiC*, *ehxA*, *etpD*, *focC*, *mcbA*, *pic*, *sepA*, *sfaD*, and *vat* were exclusive to isolates from faeces. The most frequent genes were the *fimH* and *csg*A genes (associated with adhesion proteins and the expression of extracellular surface fibers), which were found in 100% (14/14) and in 92.9% (13/14) of the *E. coli* recovered from meat samples, and in 97.9% (46/47) of the *E. coli* isolated from faeces. The *trat* and *iss* genes, related to protectins, were identified in 78.6% (11/14) and 64.3% (9/14) of the *E. coli* isolated from meat, and in 59.6% (28/47) and 53.2% (25/47) of the *E. coli* isolated from faeces. Over 92.0% of the isolates harboured fimbriae- and metal-acquisition-related genes (such as *nlpl*, *terC*, and *yeh*ABCD). The *ompT* gene, associated with the enzyme responsible for the degradation of antimicrobial peptides, was observed in 71.4% and 55.3% of the *E. coli* isolated from meat and faeces, respectively. The *astA* gene, linked to the heat-stable enterotoxin EAST-1, was identified in 14.3% (2/14) of the *E. coli* isolated from meat and in 23.4% (11/47) of the faeces isolates (Supplementary Table S1).

Concerning *Salmonella*, a high diversity of virulence factors was found, with 95 different genes identified, belonging to 10 classes (Supplementary Table S2). Specifically, 79 genes were found in *S.* London (Se_CC10), 78 genes in monophasic *Salmonella* Typhimurium (Se_CC26), and 75 genes in *S.* Typhimurium (Se_CC47) (Supplementary Table S2). Sixty genes (63.2%) were common to the three isolates, including the *invACEDJ* and *hilACD* genes (related to the secretion system group), and the *csgG* gene (related to fimbrial adherence). The *ibeB* gene, related to invasion, was identified in the Se_CC26 isolate, while the *spvB* gene, related to the enterotoxin SpvB, was found in the Se_CC47 isolate. Notably, the enterotoxin (*stn*) gene was not detected in any of the serovars.

### 2.4. Antimicrobial Resistance and Plasmids

**Figure 1:**
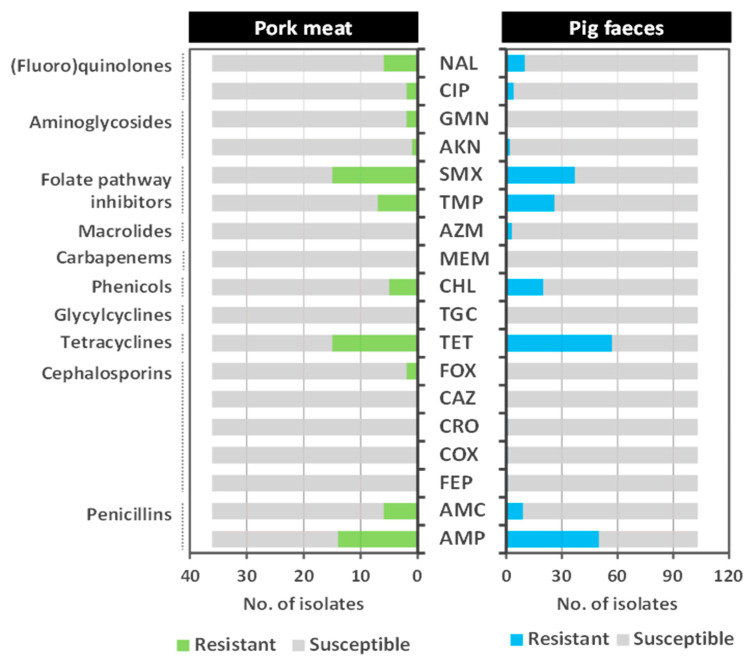
Antimicrobial resistance observed for the 139 *E. coli* isolated from raw pork meat (*n* = 36) and from pig faeces (*n* = 103) samples against a panel of 18 antibiotics. AMP, ampicillin; AMC, amoxicillin–clavulanic acid; FEP, cefepime; COX, cefotaxime; CRO, ceftriaxone; CAZ, ceftazidime; FOX, cefoxitin; TET, tetracycline; TGC, tigecycline; CHL, chloramphenicol; MEM, meropenem; AZM azithromycin; TMP, trimethoprim; SMX, sulfamethoxazole; AKN, amikacin; GEN, gentamicin; CIP, ciprofloxacin; NAL, nalidixic acid.

The AST results of the 139 *E. coli* isolates are shown in Figure 1. An overview of all the *E. coli* profiles is provided in Supplementary Table S3.

Overall, 72.7% (101/139) of the *E. coli* isolates showed resistance to at least one of the 18 tested antimicrobial agents with 75.0% (27/36) of the isolates being recovered from meat and 71.8% (74/103) from faeces samples (Supplementary Table S3).

Resistance to tetracycline (51.8%; 72/139), ampicillin (46.0%; 64/139), and sulfamethoxazole (37.4%; 52/139) were the most frequent in both types of samples (Figure 1, Supplementary Table S3). In addition, 60.4% (84/139) of the isolates showed resistance to beta-lactams, while 15.8% (22/139) exhibited resistance to (fluoro)quinolones. Although, at lower rates, resistance to the third (1.9%; 2/103) and fourth (1.0%; 1/103) generation of cephalosporins, as well as to azithromycin (2.9%; 3/103), was observed in *E. coli* recovered from faeces, while resistance to gentamicin was identified in two isolates from pork meat (5.6%; 2/36). All *E. coli* isolates were susceptible to ceftazidime, meropenem, and tigecycline. No statistically significant differences were found in the resistance to any of the antibiotics tested between *E. coli* strains isolated from meat or faecal samples (*p* > 0.05).

A total of 49 resistant *E. coli* isolates exhibited an MDR profile to three (*n* = 25), four (*n* = 22), and five (*n* = 2) antibiotic classes, of which 22.4% (11/49) were recovered from meat and 77.6% (38/49) from faeces (Supplementary Table S3). Among the MDR isolates recovered from pork meat, seven different profiles were identified, with AMP-TET-SMX being the most common (36.3%, 4/11), followed by AMP-AMC-TET-CHL-TMP-SMX (18.2%; 2/11) (Supplementary Table S3). In the MDR isolates from faeces, 20 profiles were identified, with the most frequent being AMP-TET-TMP-SMX (15.8%; 6/38) (Supplementary Table S3). Notably, two *E. coli* isolates were resistant to five antibiotic classes: one recovered from meat showed resistance to beta-lactams, tetracyclines, phenicols, folate pathway inhibitors, and quinolones (AMP-TET-CHL-TMP-SMX-NAL), and the other to beta-lactams, tetracyclines, phenicols, folate pathway inhibitors, and (fluoro)quinolones (AMP-TET-CHL-TMP-SMX-CIP).

Regarding pathogenic *E. coli*, specifically ExPEC and/or IPEC, 80.3% (49/61) were found to be MDR. Among the six IPEC strains isolated from faeces, one STEC was MDR, one was susceptible, and three of the ETEC isolates were resistant to tetracycline. One of them was susceptible to all antimicrobials tested (Supplementary Table S1).

A genomic analysis of the 61 sequenced *E. coli* isolates revealed a high diversity of antimicrobial resistance genes (ARGs), which are detailed in Table 3 and Supplementary Table S1. In total, 31 ARGs were identified, including some point mutations, conferring resistance to nine antimicrobial classes.

Several acquired resistance genes were identified in *E. coli* isolates from meat and faeces. These genes encode resistance to different antimicrobial classes, including aminoglycosides, beta-lactams, fluoro(quinolones), macrolides, phenicols, sulfonamide, tetracycline, and trimethoprim (Table 3, Supplementary Table S1). 

ARGs to beta-lactams were identified in all *E. coli* isolates resistant to ampicillin and in one susceptible isolate. Namely, the *bla*_TEM-1A_ and *bla*_TEM-1B_ genes were present in 80.4% of the isolates (37/46), and *bla*_TEM-1C_ in 8.7% (4/46). Additionally, the *bla*_CTX-M-1_ gene was detected in an ExPEC isolate recovered from faeces (Ec_SM150-1). This isolate also showed resistance to third- and fourth-generation cephalosporins as well as to the folate pathway inhibitors (Table 3, Supplementary Table S1). 

The genes encoding resistance to tetracycline *tet(A)* (36/61), and to sulfamethoxazole *sul3* (36/61) and *sul2* (21/61), were amongst the most frequently detected, either in *E. coli* isolated from meat or faeces (Table 3, Supplementary Table S1). 

Furthermore, we found that 36.1% (22/61) of the isolates contained the resistance gene *qnrS1* and exhibited point mutations in *gyrA* (p.S83A, p.S83L, p.D87N, and p.D87Y), *parC* (p.S80I), and *parE* (p.I529L), which resulted in a decreased susceptibility to quinolones. Among these isolates, 31.8% (7/22) exhibited a susceptible profile to (fluoro)quinolones (Table 3, Supplementary Table S1). We also identified other chromosomal point mutations on *gyrA*, *gyrB*, *acrB*, and 16S_*rrsD*, but they were not linked to specific phenotypes.

No plasmid-mediated *mcr* determinants or mutations in the chromosomal genes *pmrD*, *arnE*, *arnC*, *phoP*, *phoQ*, *mgrB*, and *acrB* were found in the *E. coli* isolates. 

Based on genomic data, 26.2% (16/61) of the isolates did not express a resistance phenotype while carrying the acquired ARGs *mef(B)*, *mph(A)*, *mph(B)*, *mph(G)*, *dfrA12*, *sul2*, and *qnrS1* (Table 3, Supplementary Table S1). In all these cases, the susceptible phenotype was confirmed. 

Resistance genes associated with other antimicrobials have been identified. Specifically, aminoglycoside resistance genes encoding for streptomycin (*aph(3*″*)-Ib*, and *aph(6)-Id)* and/or streptomycin/spectinomycin (*aadA1*, *aadA2*, *aadA5*, and *aadA12)* were identified in 72.1% (44/61) of the isolates. Among these, 77.3% (34/44) were isolated from faeces (Table 3, Supplementary Table S1). Additionally, one isolate (Ec_CC8-3) was found to carry the *qacL* gene, a biocide resistance gene belonging to the disinfectant and antiseptic class that confers resistance to quaternary ammonium compounds.

Regarding *Salmonella* spp., all three isolates were resistant to at least two of the tested antimicrobials. Whereas Se_CC10 isolate was resistant to (fluoro)quinolones, both Se_CC26 and Se_CC47 were considered to be MDR (Table 3, Supplementary Table S1). In total, we identified 13 ARGs, linked to resistance to nine antimicrobial classes. The genotypes of the identified ARGs confirmed the phenotypic profiles of the three isolates. Additionally, we detected genes encoding resistance to aminoglycosides, namely, the cryptic *Salmonella aac(6*′*)-Iaa* gene.

Several plasmid replicons were identified in *E. coli* isolates (Supplementary Table S1). The most prevalent replicons were IncFIB (57.1% and 59.6%, in meat and faeces isolates, respectively) and IncFII (50.0% and 46.8%, in meat and faeces isolates, respectively). The ESBL-producing *E. coli* (Ec_SM150-1) harboured the IncFIA, IncFIB(AP001918), IncFII(29), and IncI1-I(Alpha) plasmids. For *Salmonella* isolates, six different plasmids were identified, with each isolate carrying one to three replicons (Supplementary Table S1).

### 2.5. Phylogenetic Analysis

**Figure 2:**
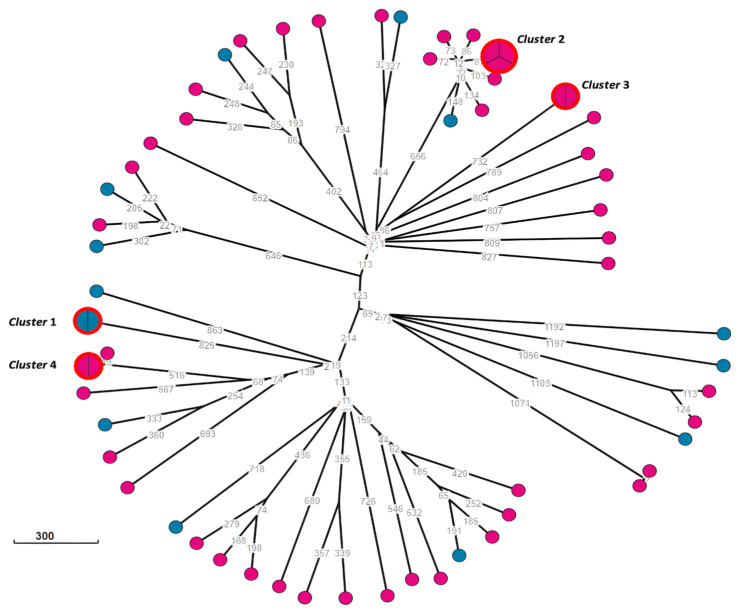
Phylogenetic analysis (minimum spanning tree) of the 14 *E. coli* isolates from meat samples (round pink circles) and the 47 *E. coli* isolates from faecal samples (round blue circles) using the cgMLST V1 + HierCC V1 scheme with 2513 loci available on the Enterobase platform (*n* = 103). The numbers shown on the branches represent allelic differences between the isolates. Clusters formed with ≤7 allelic differences between isolates are highlighted in red. The size of the clusters is proportional to the number of isolates included.

To assess the genetic relationship between the 14 *E. coli* isolates from food matrices and the 47 isolates from animal matrices, a minimum spanning tree based on the cgMLST was carried out (Figure 2).

The cgMLST analysis of the 61 *E. coli* isolates (Figure 2) identified four clusters, which included nine ExPEC from four different STs (Supplementary Table S1). Cluster 1 grouped two isolates from meat samples (Ec_CC27-4 and Ec_CC28-2), while clusters 2 (Ec_SM145-1, Ec_SM146-1 and Ec_SM148-1), 3 (Ec_SM1-1 and Ec_SM196-1), and 4 (Ec_SM44-3 and Ec_SM79-1) only included isolates from faeces samples. Each cluster showed complete agreement in terms of the phylogroup, ST, serotype, virulence genes, and resistance genes among the *E. coli* isolates. Clusters 1, 2, and 4 comprised isolates from samples belonging to the same meat producer (producer 1) or pig farm (cluster 2—farm A; and cluster 4—farm J). Cluster 3 consisted of two *E. coli* isolates from faeces samples from different farms (farms A and Q) (Supplementary Table S1).

Regarding *Salmonella* spp. isolates from meat samples, a minimum spanning tree was created using the cgMLST V2 + HierCC V1 scheme (with 3002 loci) The analysis included 28 *S.* London strains isolated in 2023: 16 from France, 7 from the United States of unknown source, 3 from Portugal (one clinical isolate, one isolate of unknown source, and the meat isolate from this study), and 2 clinical isolates from Canada. Two clusters were identified, but the isolate under investigation (Se_CC10) was not part of any cluster. In addition, a phylogenetic tree was generated using 879 international and national clinical, food, and animal isolates of *S.* Typhimurium and monophasic *S.* Typhimurium isolated in 2023. However, the isolates Se_CC26 and Se_CC47 were not included in any cluster.

## 3. Discussion

Foodborne infections have become increasingly common over the years and now pose a serious public health problem globally, causing a significant economic and health burden. One of the main sources of these diseases is food-producing animals, which can contaminate derived food products with pathogenic and antimicrobial-resistant bacteria, namely, *E. coli* and *Salmonella* spp.

In this study, we analysed 100 faecal samples of Portuguese fattening pigs raised for human consumption, collected from different slaughterhouses, and 52 samples of Portuguese-origin raw pork meat, acquired independently at several retail stores. The microbiological quality of the meat samples was evaluated for the presence of *E. coli* and *Salmonella* spp. Additionally, isolates of both species were further characterised phenotypically and genotypically. 

*E. coli* was found in all faecal samples, which is not surprising as *E. coli* is a commensal in pig intestinal microbiota [29]. Although several studies have reported the presence of *Salmonella* spp. in pig faeces, with rates of 47.5% in Thailand [30], and 52.4% in Spain [31], or in pig carcasses collected in a slaughterhouse environment with rates of 21.0% [8] and 7.3% [32] in Portugal, and 11.5% in Ireland [33], in the present study, *Salmonella* spp. was not detected. Despite no control programmes to reduce the prevalence of *Salmonella* spp. in pig farms currently being implemented in Portugal, the observed absence in pig faeces may result from several factors, such as the intermittent rather than continuous shedding, or even the low shedding levels of bacteria, which can render the microbiological detection unreliable and affect the final detection rate. To corroborate our findings, it would be advantageous to evaluate the prevalence at on-farm suppliers and during pigs’ transportation and lairage, as well as to analyse other pig samples, such as lymph nodes. 

Regarding raw pork meat samples, 69.2% tested positive for *E. coli*, which is consistent with similar studies conducted in Thailand (68.0%) [29], Nepal (60.0%) [34], and Mexico (57.7%) [35]. On the other hand, the prevalence of *Salmonella* spp. (5.8%) was similar to rates reported in Brazil (6.3%) [36] and in Italy (5.7%) [37], but lower when compared to those found in China (73.1%) [38] and Romania (22.6%) [39]. Considering the microbiological quality parameters defined in this study, four out of the 52 raw pork meat samples were classified as unsatisfactory for *E. coli* levels, and three for the presence of *Salmonella* spp. As far as we know, studies of the microbiological quality evaluation of *E. coli* and *Salmonella* spp. in non-minced raw pork meat in Portugal are scarce. A study carried out in the northern region of Portugal assessed the microbiological quality of packaged meat approaching the end of its shelf life, and all the pork meat samples were found to be satisfactory for the presence of these bacteria [40]. Similarly, a German study found that all the analysed raw pork meat samples met satisfactory parameters for *E. coli* [41].

In the present study, the detection of *E. coli* (IPEC and/or ExPEC) and *Salmonella* spp. suggests potential issues with quality management, which may be associated with non-compliance with good hygiene practices in the production, distribution, and retail of pork meat [35]. Although there is no correlation between our meat and faecal samples (as they were independently collected from each other), the process of cutting and preparing raw meat in commercial retail stores can lead to contamination and lower microbiological quality, posing a significant public health risk. Although heat treatment can inactivate *E. coli* and *Salmonella* spp. in food products, inadequate meat preparation, poor hygiene, and cross-contamination between raw and cooked foods, as well as between surfaces that contact with infected raw meat, can still contribute to the spread of these bacteria [42,43].

The identification of pathogenic *E. coli* was based on the PCR and/or WGS results. While no IPECs were identified in the pork meat isolates, 2.0% of the isolates in the pig faeces samples were identified as STEC and 4.0% as ETEC. The STEC isolates carried the *stx2e* gene but not the *eae* gene, as reported in 18.5% of STEC infections in the EU in 2022 [44]. The *stx2e* variant of Shiga toxin has been identified in several studies, including those conducted in pigs produced for human consumption, in pork and pork products [45,46,47,48]. The *stx2e* subtype is frequently associated with oedema disease in pigs but can also be detected in asymptomatic carriers [45]. Both STEC isolates in this study belonged to serogroup O8, which is not one of the most frequently identified in Europe [6]. Of note, this serogroup was found in *E. coli* isolated from meat (14.3%) and from pig faeces (14.9%) and was associated with STEC and ExPEC pathotypes. This reinforces the idea that the serogroup alone is not a reliable indicator of the degree of pathogenicity of *E. coli*. Among the ETEC isolates identified in pig faecal samples, the *stb* gene, which encodes a heat-stable enterotoxin frequently identified in ETEC of porcine origin, was detected in association with the *astA* gene that encodes the heat-stable enterotoxin EAST-1. This association has been demonstrated to increase the virulence of ETEC isolates [49]. Although no IPEC was found in the pork meat samples, it is important to note that the presence of these bacteria in the slaughterhouse environment can be a source of contamination for products of animal origin, especially when the hygiene conditions are not maintained. Bacteria from the intestinal contents of the carcass can transfer to the carcasses along the slaughter line, leading to contamination of the final food product. The pathogenic potential of *E. coli* is associated with the presence and expression of several virulence genes. Interestingly, all the *E. coli* isolates sequenced in this study (14 isolates from meat and 47 isolates from faeces) contained virulence genes associated with ExPEC. These genes were also identified in all the isolates classified as IPEC by PCR. The ExPEC virulence factors belonging to the adhesins class, encoded by the *csgA* (found in 100% of meat isolates and in 97.9% of pig faecal isolates) and *fimH* (found in 92.9% of meat isolates and in 97.9% of pig faecal isolates) were the most frequent. These findings are consistent with those reported by other studies [50,51,52]. Other virulence determinants frequently identified were as follows: *traT* (78.6% in meat isolates and 59.6% in pig faeces isolates) and *iss* (64.3% in meat isolates and 53.2% in pig faeces isolates), both of which are genes encoding membrane proteins; *gad* (71.4% in meat isolates and 89.4% in pig faeces isolates), encoding the glutamic acid decarboxylase enzyme; and *terC* (100% in meat and pig faeces isolates), that encodes the tellurium ion resistance protein. These genes have also been frequently reported by several studies [50,51,52]. The high prevalence of strains (90.2%) harbouring virulence genes associated with the ExPEC pathotype is relevant. These results support the previously proposed hypothesis [52,53,54,55] that ExPEC could be considered opportunistic microorganisms, since they are part of the intestinal flora of humans and animals, and only show their virulence potential when they colonise extraintestinal tissues. Nevertheless, further studies are required to gain a better understanding of the virulence factors of commensal *E. coli* and their role in extraintestinal infections.

The 61 sequenced *E. coli* isolates were further categorised into eight phylogroups (A, B1, B2, C, D, E, F, and G), according to the Clermont classification [55]. Despite the high diversity, most of the strains were classified as belonging to phylogroups A and B1. These phylogroups are commonly associated with commensal *E. coli*, but they may also be related to IPEC [56]. Due to the ubiquity of this bacterium, the association between the phylogroup and the pathogenicity of *E. coli* is challenging to establish [56].

In the present study, three different *Salmonella* spp. serotypes were found in the samples of pig meat: *S.* London, *S.* Typhimurium, and *S.* monophasic Typhimurium. Among these, *S.* Typhimurium and monophasic Typhimurium are two of the most prevalent serotypes in Europe and are mainly associated with pigs [6]. Although *S.* London is less prevalent in Europe, it has been widely reported in China [57]. In Portugal, these serotypes have been previously identified in the environment of pig slaughterhouses [8,11]. Several studies have highlighted the role of specific genes such as *invA*, *stn*, *hilA*, *spvABCD*, and *spvR* in the pathogenicity of non-typhoidal *Salmonella*, particularly in terms of host cell interaction, enterotoxin production, and invasion [58]. In the present study, the *hilA* gene was identified in the three isolates, while the *spvB* gene was identified only in *S.* Typhimurium. The *invA* gene, which is considered a genetic marker for most serotypes of *Salmonella* spp. [59], was observed in the three isolates. Moreover, the *spvC*, *spvR*, *stn,* and *fimA* genes were not identified in none. Despite the limited number of isolates in this study, the findings are aligned with existing literature and emphasise the importance of evaluating these genomic determinants in *Salmonella* spp. isolated from food matrices, given their zoonotic potential [60].

A total of 43 different STs were identified among the *E. coli* isolates. Of these, 13 STs were observed in the meat isolates, including three new STs, and 30 STs were identified in the pig faeces isolates; out of these, 17.0% belonged to ST101. One ExPEC isolated from meat (CC47) belonged to ST131, which is an emerging multidrug-resistant lineage among ExPEC isolates worldwide. This virulent clone also presents a high adaptability to different hosts, including humans, food-producing animals, wild and companion animals, and retail meat [51,52], and has been associated with several resistance determinants, including ESBL production and fluoroquinolone resistances [61]. In fact, the ST131 isolate in this study (CC47) revealed an MDR profile to ampicillin, tetracycline, nalidixic acid, chloramphenicol, trimethoprim, and sulfamethoxazole, but was not an ESBL-producing *E. coli*. The resistance to fluoroquinolones was associated with amino acid substitutions within the quinolone-resistance-determining regions (QRDR) of *gyrA* (p.S83A; p.S83L; p.D87N; p.D87Y) and *parE* (p.I529L). Furthermore, three common STs were identified among *E. coli* isolated from meat and pig faeces: ST10, ST88, and ST101. Previous studies demonstrated that ST10 and ST88 are globally distributed in animals, namely, in pigs, as well as in humans, suggesting the potential circulation of these STs between animals and humans through the consumption of contaminated food of animal origin [62]. For *Salmonella* spp. isolates, the identified STs were ST155 (*S.* London), ST34 (*S.* monophasic Typhimurium), and ST19 (*S.* Typhimurium), which are predominant pandemic genotypes associated with several foodborne outbreaks [6]. 

Antimicrobial resistance and antimicrobial-resistant microorganisms are considered to be among the main public health and global threats worldwide. Antimicrobial use in food-producing animals has been implicated in the emergence and spread of MDR microorganisms through the food chain [63]. There is particular concern about the presence of ARGs related to antibiotics of critical importance for human and animal health, namely, fluoro(quinolones), macrolides, and cephalosporins. Point mutations in the coding region of the enzymes DNA gyrase and DNA topoisomerase IV (*gyrA*, *parC,* and *parE* genes, respectively) and the identification of the plasmid *qnr* genes (*qnrS1*) are associated with predictive resistance to fluoro(quinolones) [64].

In the present study, 75.0% of the *E. coli* and 66.7% of the *Salmonella* spp. isolates from meat, and 71.8% of the *E. coli* isolates from pig faeces were phenotypically resistant. These findings are consistent with those reported by others in Portugal [25], where the proportion of antibiotic-resistant *E. coli* and *Salmonella* spp. was also high (100% and 78.5%, respectively). *E. coli* resistance to tetracycline (41.7% in meat and 55.3% in pig faeces isolates), ampicillin (38.9% in meat and 48.5% in pig faeces isolates), and sulfamethoxazole (41.7% in meat and 35.9% in pig faeces isolates) were the most frequent. Resistance to third and fourth-generation cephalosporins was low (between 0.0–1.0%). For *Salmonella* spp., despite the low number of isolates, resistance to ampicillin and to sulfamethoxazole were the most common, aligning with data from the latest annual surveillance report on antimicrobial resistance in zoonotic bacteria in the EU [64]. As reported by other studies [12,63], all the isolates exhibited susceptibility to ceftazidime, meropenem, and tigecycline. The inappropriate and abusive use of antibiotics in pig productions can contribute to the high resistance rates of *E. coli* and *Salmonella* spp. isolates [26]. In Portugal, like most EU countries, tetracyclines, penicillins, and sulfonamides present the highest sales volumes for veterinary medical purposes [27]. An MDR profile was observed in 49 *E. coli*, with 40.7% recovered from meat and 51.4% recovered from pig faeces. The frequency of MDR isolates found is higher than that reported by the EFSA/ECDC report, which showed values of 34.2% for *E. coli* isolated from pigs produced for human consumption [6]. As previously reported [63], the most prevalent profile observed was AMP-TET-SMX-TMP. Nevertheless, caution is advised when making such comparisons, since biases stemming from the type of tests and interpretation criteria used, as well as the origin of the data (national surveillance programmes, and studies on farms or in slaughterhouses), can be introduced.

Based on the WGS findings, a high diversity of ARGs and point mutations that predict the resistance to nine antimicrobial classes were identified. Regarding *E. coli*, the genes *tet(A)*, *sul2*, *sul3*, *dfrA1,* and *dfrA12* were among the most frequently identified ARGs in isolates from faeces and meat [50,52,63]. In addition, genes predictive of the resistance to aminoglycosides (*aadA1*, *addA2*, *addA2b*, *aadA5*, *aadA12*, *aac(6*′*)-Iaa*, *aph(3*″*)-Ib*, and *aph(6)- Id)*, phenicols (*catA2*, *cml*, *cmlA1*, and *floR*), and macrolides (*mef(B)*, *mph(A)*, *mph(B)*, and *mph(G)*) were also found. The most frequent ARG to beta-lactamases were *bla*_TEM-1A_, *bla*_TEM-1B_, and *bla*_TEM-1C_ (80.4%). Overall, only one ESBL-producing *E. coli* (*bla*_CTX-M-1_) was detected. The *bla*_CTX-M-1_ gene is commonly reported in isolates from fattening pigs and pig meat in Europe [6] and was also previously detected in Portugal [61]. In this study, the prevalence of ESBL-producing *E. coli* was very low, despite the high rates previously reported in Portuguese studies in food-producing animals (pigs: 49.0%; cattle: 7.0%; and turkeys: 4.0%) and in pork meat (10.5%) [61,65]. This finding is in line with data from the most recent EFSA/ECDC report on antimicrobial resistance in *E. coli* from food-producing animals [64]. Nevertheless, it is important to note that not all isolates were selected for WGS, so there may be other ESBL-producing *E. coli* in this study. 

Although several Portuguese studies have shown the presence of colistin resistance genes (*mcr* genes) in *E. coli* and *Salmonella* spp. isolated from food-producing animals, including pigs and different food products [64,65,66], in this work, no *mcr* genes were detected in either species, which could be attributed to a more restricted and conscientious use in veterinary medicine of this important last-resort antibiotic. Food-producing animals can be important reservoirs of *E. coli* and *Salmonella* spp. carrying resistance genes, which can be transmitted to humans by direct contact or through the food chain if proper hygiene practices are not followed. A study conducted in Germany [41] showed that pork meat can be a reservoir of ESBL, even when the microbiological quality parameters (such as *Enterobacteriaceae*, *E. coli*, and mesophilic aerobic microorganism counts) are within satisfactory limits. However, due to the small number of *Salmonella* spp. isolates in this study, it is challenging to discuss the results. 

In this study, IncF and IncX were the most frequently identified plasmids. IncF plasmids are prevalent in Europe and have been identified in *E. coli* isolated from humans, animals, and the environment [67,68]. These plasmids are known for carrying genes that provide resistance to various classes of antibiotics, including penicillins, carbapenems, aminoglycosides, quinolones, sulfonamides, and tetracyclines [67,68]. On the other hand, IncX plasmids are predominant in *Salmonella* spp. and *E. coli* isolates for both human and animal sources, and usually contain resistance genes for quinolones, tetracyclines, and trimethoprim [67]. Additionally, the *bla*_CTX-M-1_ gene is frequently described in plasmids from the IncI1-l group [51,67], as observed in the present study. 

The phylogenetic analysis revealed that the three *Salmonella* isolates did not integrate any cluster. However, four clusters of *E. coli* isolates were identified. Cluster 1 included isolates from samples belonging to the same brand, meat producer, and pig farm, suggesting that contamination may have occurred at the primary production stage or at the slaughterhouse, probably during the evisceration process [3]. Clusters 2 and 4 included isolates from faecal samples belonging to the same pig farm, while Cluster 3 comprised *E. coli* isolates from pig faeces from two different farms collected at different times. Due to the lack of data and information about the potential link between the two farms, we cannot draw conclusions about the phylogenetic relationship between these *E. coli* isolates.

While beyond the scope of the present study, a future analysis of siderophore-microcins producing *E. coli* and *Salmonella* genes may prove relevant, as these antimicrobial peptides are considered to be a key determinant of bacterial competition within the intestinal niche [68]. In parallel, a more detailed analysis of siderophores, which are thought to be secondary metabolites that capture iron and promote bacterial growth and development, should be considered [69,70].

## 4. Materials and Methods

### 4.1. Sample Collection

In the present study, 152 samples of animal and food origin were evaluated.

Between November 2022 and March 2023, 100 faecal samples from fattening pigs raised in Portugal for human consumption were collected at different slaughterhouses. Faecal samples were collected directly from the rectum after evisceration, on a convenience basis. The samples were transported under refrigerated conditions and immediately processed or kept refrigerated at −20 °C.

Moreover, 52 raw pork meat samples (e.g., leg and loin steaks, spare ribs, rib chops, tenderloin, and loin dices) of Portuguese origin were independently obtained from several retail stores between January and April 2023. All samples were kept refrigerated (4 °C) during transportation and processed within 24 h after purchase, before their expiration date. All sample packaging was disinfected with 70% ethanol before being processed.

### 4.2. Isolation Methodology from Faeces

*E. coli* and *Salmonella* spp. were isolated as previously described [71]. For *E. coli*, samples underwent pre-enrichment in buffered peptone water (BPW, Oxoid, Basingstoke, Hampshire, UK) and were then plated on Hektoen agar (HK, Biokar Diagnostics, Pantin, France). Up to six colonies with characteristic morphology were selected and sub-cultured on Tryptone Soy Agar (TSA, Biokar Diagnostics, Pantin, France). Suspicious colonies were confirmed by biochemical identification on the VITEK compact system (bioMérieux, Marcy L’Etoile, France) or by *E. coli 16S* rRNA PCR [72] (Supplementary Table S4). For *Salmonella* spp., isolation was based on ISO 6579-1:2017 [73], as previously described [71]. Following non-selective and selective enrichment, presumptive colonies were isolated on TSA and identified using the VITEK 2 compact system. 

All positive isolates were stored at −80 °C in Tryptone Soy Broth (TSB, Biokar Diagnostics, Pantin, France) with 20% glycerol.

### 4.3. Microbiological and Quality Analysis from Meat

All microbiological analysis and microbiological evaluations were performed according to the general requirements and guidance described in ISO 7218:2007 [74]. 

The detection and enumeration of *E. coli* was performed using the TEMPO^®^ EC automated most probable number system (bioMérieux, Marcy L’Etoile, France), according to the manufacturer’s instructions. The remaining mixture was then incubated at 37 ± 1 °C for 18–24 h for subsequent detection of *Salmonella* spp. and isolation of *E. coli*. 

For *Salmonella* spp., 0.1 mL of the incubated mixture was transferred into 10 mL of Salmonella Xpress 2 broth (SX2, bioMérieux, Marcy L’Etoile, France) and incubated at 41.5 ± 1 °C for 18–24 h. Simultaneously, 500 µL was tested using the VIDAS^®^ Easy SLM (bioMérieux, Marcy L’Etoile, France) system, according to the manufacturer’s instructions. All positive samples were plated on both IRIS agar (Biokar Diagnostics, Pantin, France) and Xylose Lysine Deoxycholate agar (XLD, bioMérieux, Marcy L’Etoile, France), and incubated at 37 ± 1 °C for 18–24 h [73]. For *E. coli* isolation, a loopful of the same incubated mixture was streaked on the surface of Chromogenic Coliform Agar (CCA, Biokar Diagnostics, Pantin, France) and incubated at 37 ± 1 °C, during 18–24 h. The identification of presumptive *E. coli* and *Salmonella* spp. isolates were confirmed using the VITEK^®^2 compact system (bioMérieux, Marcy L’Etoile, France) or by *E. coli 16S* rRNA PCR [72] (Supplementary Table S4). All positive isolates were stored at −80 °C in TSB with 20% glycerol.

The microbiological quality was analysed according to the criteria presented in Table 4, based on the European regulation [28] for process hygiene criteria for meat and products thereof: number of colony-forming units *per* gram (cfu/g) for *E. coli* (“meat preparations”) and presence or absence of *Salmonella* spp. (“minced meat and meat preparations made from other species than poultry intended to be eaten cooked”).

### 4.4. Bacterial Typing and Serotyping

Identification of potential intestinal pathogenic *E. coli* was characterised by multiplex PCR targeting specific virulence genes (*eae*, *aggR*, *aatA*, *aaiC*, *elt*, *est*, and *ipaH*) modified from [75,76,77] and for the presence of Shiga toxins *stx1* and *stx2* [78], as previously described [71]. Pools containing a maximum of six colonies from each sample were initially tested and were subsequently individualised when a PCR-positive result was obtained. PCR profiles and primer sequences are presented in the Supplementary Table S4. IPEC pathotype was defined when one or more of the tested genes was detected.

*Salmonella* serotyping was achieved by the slide agglutination method for somatic and flagellar antigens (SSI Diagnostica, Hillerod, Denmark; and Sifin diagnostics, Berlin, Germany), according to the Kauffmann–White–Le Minor scheme [79].

### 4.5. Antimicrobial Susceptibility Testing

Antimicrobial susceptibility was evaluated by disk-diffusion, following the European Committee on Antimicrobial Susceptibility Testing (EUCAST) recommendations [80], using a panel of 18 antibiotics: amikacin (AKN, 30 μg), ampicillin (AMP, 10 μg), amoxicillin–clavulanic Acid (AMC, 30 μg), azithromycin (AZM, 15 μg), cefepime (FEP, 30 μg), cefotaxime (COX, 5 μg), cefoxitin (FOX, 30 μg), ceftazidime (CAZ, 10 μg), ceftriaxone (CRO, 30 μg), chloramphenicol (CHL, 30 μg), ciprofloxacin (CIP, 5 μg) for *E. coli* and pefloxacin (PEF, 5 μg) for *Salmonella* spp., gentamicin (GMN, 10 μg), meropenem (MEM, 10 μg), nalidixic Acid (NAL, 30 μg), sulfamethoxazole (SMX, 100 μg), tetracycline (TET, 30 μg), tigecycline (TGC, 15 μg), and trimethoprim (TMP, 5 μg). Interpretation of inhibition zones were performed according to the epidemiological cut-off values (ECOFFs) [80]. MDR isolates were defined when resistance to one or more antimicrobials from three or more different antimicrobial classes was observed [81].

### 4.6. Detection of mcr 1–9 Genes

PCR for *mcr* 1–9 genes was tested in all isolates using two multiplex PCR based on different protocol modifications [82,83,84], which are detailed in Supplementary Table S4. The amplification was carried out in a reaction volume of 20 µL and using the HotStar Taq master mix (Qiagen, Hilden, Germany), according to the manufacturer’s recommendations. *E. coli* NCTC 13,846 (Selectrol^®^ TCS Biosciences, Buckingham, UK), positive for *mcr-1*, and genomic characterised DNAs for *mcr-2* to -*9* genes were used as positive controls.

### 4.7. Whole-Genome Sequencing and Genomic Analysis

All strains of *Salmonella* spp., all pathogenic and/or MDR *E. coli,* and all isolates with a doubtful IPEC PCR result underwent Whole-Genome Sequencing (WGS). Following DNA extraction (ISOLATE II Genomic DNA Kit (Bioline, London, UK) and quantitation (dsDNA HS Assay Kit; Thermo Fisher Scientific, Waltham, MA, USA), high-quality DNA samples were subjected to the NexteraXT library preparation protocol (2 × 250 bp or 2 × 500 bp; Illumina, San Diego, CA, USA) and sequenced on either a MiSeq or a NextSeq instrument (Illumina, San Diego, CA, USA), according to the manufacturer’s instructions.

The raw reads were submitted to the QAssembly pipeline (v 3.61) of EnteroBase (https://enterobase.warwick.ac.uk/; accessed on 1 July 2023) for quality control, trimming, and generating assemblies of high quality. Online bioinformatic tools from EnteroBase were used to determine *in silico E. coli* phylogroup and ST. The assemblies were also submitted to the Centre for Genomic and Epidemiology (CGE, http://www.genomicepidemiology.org, accessed on 7 July 2023) to assess *in silico* resistance genes and point mutations (ResFinder 4.4.2), plasmid replicons (PlasmidFinder 2.1), ST (MLST 2.0), *E. coli* virulence genes (VirulenceFinder 2.0) and serotypes (SeroTypeFinder 2.0), and *in silico Salmonella* serotyping (SeqSero 1.2). *E. coli* resistance genes and point mutations were double-checked by the submission of raw reads. For *E. coli*, the presence of two or more ExPEC-typical virulence genes were used for the classification of this pathotype [53,54]. *Salmonella* virulence genes were obtained from the Virulence Factors of Pathogenic Bacteria (VFDB) platform (http://www.mgc.ac.cn/cgi-bin/VFs/v5/main.cgi; accessed on 9 November 2023), using the VFanalyser tool.

Comparative phylogenetic analysis was performed using the Enterobase cgMLST V1+HierCC V1 for *E. coli* (2513 *loci*) and the cgMLST V2+HierCC V1 for *Salmonella* (3002 *loci*) [85,86]. GrapeTree figures were constructed using the minimal spanning tree algorithm NINJA NJ (MSTree V2) [87].

Sequence reads were deposited in the European Nucleotide Archive (ENA) under the bioprojects PRJEB77053 (pig faeces *E. coli* isolates), PRJEB54735 (raw pork meat *E. coli* isolates), and PRJEB32515 (*Salmonella*). Accession numbers are presented in Supplementary Table S1.

## 5. Conclusions

Most foodborne diseases, caused by pathogenic bacteria, have a zoonotic origin, with food-producing animals being the main reservoirs, and animal-derived foods, such as meat, being the primary sources of transmission. Antibiotic resistance associated with many of these bacteria exacerbates the risk to human and animal health. This study characterised strains of *E. coli* and *Salmonella* spp. isolated from pig faeces, produced for human consumption, and from raw pork meat, one of the most consumed food products in Portugal. These data highlight the importance of defining specific and more comprehensive microbiological safety criteria (beyond those mentioned in current legislation) for the detection of these bacteria in pig faeces and pork meat in general.

Although pork is not a ready-to-eat food, cross-contamination during the preparation of contaminated food items can pose a risk to consumer health. The high frequency of resistant *E. coli* and *Salmonella* spp., especially in meat samples, is of particular concern for public health. While the treatment of gastrointestinal infections does not typically involve antibiotics, the resistance genes present in these bacteria can potentially limit the treatment of more severe infections and can also spread to other bacteria through mobile genetic elements.

In summary, the presence of pathogenic bacteria in pigs produced for human consumption underscores the need for better hygiene practices throughout the entire food chain. It is essential that we implement monitoring and control strategies through a One Health approach. The investment in “farm to fork” research and understanding how zoonotic bacteria persist in animal reservoirs and enter the food chain are crucial steps for implementing these strategies, for instance. Additionally, promoting biosecurity measures at the industry and retail levels and investing in consumer education and awareness are critical steps in addressing this public health challenge.

## Figures and Tables

**Table 1:** Microbiological quality of the 52 raw pork meat samples, according to the defined parameters.

Microbiological Quality	Satisfactory (%)	Borderline (%)	Unsatisfactory (%)
*E. coli* count (cfu/g)	48 (92.3)	0	4 (7.7)
Presence of *Salmonella* spp.	49 (94.2)	N/A	3 (5.8)

cfu/g, colony-forming units *per* gram; N/A, not applicable.

**Table 2:** Isolation and characterisation of *E. coli* and *Salmonella* spp. in the 152 tested samples, according to the PCR and/or WGS results.

No. of Tested Samples		Pork Meat	Pig Faeces	Total
		52	100	152
*E. coli* isolates	Total (% +ve)	36 (69.2)	100 (100)	136 (89.5)
	Pathogenic (% +ve)	14 (26.9)	47 (47.0 ^a^)	61 (40.1)
	STEC (% +ve)	0	2 (2.0 ^b^)	2 (1.3)
	ETEC (% +ve)	0	4 (4.0 ^c^)	4 (2.6)
	ExPEC (% +ve)	14 (26.9)	41 (41.0 ^b,c^)	55 (36.2)
*Salmonella* isolates	Total (% +ve)	3 (5.8)	0	3 (2.0)
	*S.* London (% +ve)	1 (1.9)	0	1 (0.7)
	*S.* Typhimurium (% +ve)	1 (1.9)	0	1 (0.7)
	*S.* monophasic Typhimurium (% +ve)	1 (1.9)	0	1 (0.7)

STEC, Shiga-toxin-producing *E. coli*; ETEC, enterotoxigenic *E. coli*; ExPEC, extraintestinal pathogenic *E. coli*; *S*. *Salmonella*; No., number; +ve, positive samples; ^a^ The data presented correspond to the number of studied isolates; ^b^ Different pathogenic *E. coli* were detected in the same sample (sample SM164): one isolate was identified as STEC (Ec_SM164-1) and the other as ExPEC (Ec_SM164-3); ^c^ Different pathogenic *E. coli* were detected in the same sample (samples SM8 and SM43): in each sample, one isolate was identified as ETEC (Ec_SM8-1 and Ec_SM43-5) and the other as ExPEC (Ec_SM8-3 and Ec_SM43-2) (Supplementary Table S1).

**Table 3:** Phenotypic and genotypic antimicrobial characterisation of the 64 sequenced isolates.

***E. coli***			
**Isolate ID**	**Phenotypic profile**	**Resistance genes and point mutations related to phenotype profile**	**Other resistance genes**
Ec_SM1-1	AMP, AMC, TET, CHL, TMP, SMX	*bla*_TEM-1C_, *tet(A)*, *floR*, *dfrA14*, *sul2*	*aph(3*″*)-Ib*, *aph(6)-Id*
Ec_SM3-1	AMP, TET, CHL, TMP	*bla*_TEM-1B_, *tet(A)*, *cmlA1*, *dfrA12*	*aadA1*, *aadA2*
Ec_SM5-1	AMP, TET, TMP, SMX	*bla*_TEM-1B_, *tet(B)*, *dfrA1*, *sul3*,	*aadA1*
Ec_SM6-1	AMP, TET, TMP, SMX	*bla*_TEM-1B_, *tet(A)*, *dfrA1*, *sul3*	*qnrS1*, *aadA1*, *aph(6)-Id*
Ec_SM8-1	Susceptible	No	No
Ec_SM8-3	TET, CHL, TMP, SMX	*tet(A)*, *cmlA1*, *dfrA12*, *sul3*	*aadA1*, *aadA2*, *qacL*
Ec_SM10-1	TET	*tet(A)*	No
Ec_SM11-2	AMP, TET, TMP, SMX	*bla*_TEM-1B_, *tet(A)*, *dfrA1*, *sul3*	*aadA1*
Ec_SM12-2	TET, CHL, TMP, SMX	*tet(A)*, *cmlA1*, *dfrA12*, *sul3*	*mef(B)*, *aadA1*, *aadA2*
Ec_SM13-3	AMP, TET, SMX	*bla*_TEM-1B_, *tet(B)*, *sul3*	No
Ec_SM15-3	AMP, TET, CHL	*bla*_TEM-1B_, *tet(B)*, *floR*	*sul2*, *aph(3*″*)-Ib*, *aph(6)-Id*
Ec_SM16-2	AMP, TET, CHL, TMP	*bla*_TEM-1B_, *tet(A)*, *cmlA1*, *dfrA12*	*aadA2*
Ec_SM17-4	AMP, AMC, TET, CHL	*bla*_TEM-1B_, *tet(B)*, *floR*	*aph(6)-Id*
Ec_SM19-4	AMP, AMC, TET, CHL, TMP, SMX	*bla*_TEM-1B_, *tet(A)*, *cmlA1*, *dfrA12*, *sul3*	*aadA1*, *aadA2*
Ec_SM24-1	AMP, TET, SMX, NAL, CIP	*bla*_TEM-1B_, *tet(B)*, *sul3*, *qnrS1*, *gyrA* (p.S83A)	*aph(3*″*)-Ib*, *aph(6)-Id*
Ec_SM28-3	AMP, AMC, TET, TMP, SMX	*bla*_TEM-1A_, *tet(A)*, *dfrA1*, *sul3*	No
Ec_SM30-2	TET	*tet(B)*	No
Ec_SM31-3	AMP, TET, TMP, SMX, NAL	*bla*_TEM-1B_, *tet(A)*, *dfrA14*, *sul2*, *gyrA* (p.D87Y)	*mph(G)*, *aph(6)-Id*
Ec_SM33-4	AMP, CHL, SMX	*bla*_TEM-1B_, *floR*, *sul2*	*aph(3*″*)-Ib*, *aph(6)-Id*
Ec_SM34-2	TET	*tet(A)*	No
Ec_SM39-3	Susceptible	No	No
Ec_SM43-2	AMP, AMC, TET, TMP, SMX	*bla*_TEM-1B_, *tet(B)*, *dfrA1*, *sul1*	*aadA1*
Ec_SM43-5	TET	*tet(A)*	No
Ec_SM44-3	AMP, TET, SMX, NAL	*bla*_TEM-1B_, *tet(A)*, *sul3*, *gyrA* (p.S83A)	*mef(B)*, *aph(6)-Id*
Ec_SM45-4	AMP, TET, CHL, TMP, SMX	*bla*_TEM-1B_, *tet(B)*, *cmlA1*, *dfrA12*, *sul3*	*aadA1*, *aadA2*, *aph(6)-Id*
Ec_SM73-1	AMP, TET, CHL, SMX	*bla*_TEM-1B_, *tet(A)*, *cmlA1*, *sul2*, *sul3*	*qnrS1*, *dfrA12*, *aadA1*
Ec_SM74-1	AMP, TET, CHL, SMX	*bla*_TEM-1B_, *tet(A)*, *floR*, *sul2*	*qnrS1*
Ec_SM77-1	AMP, TET, TMP, SMX, NAL, CIP	*bla*_TEM-1B_, *tet(A)*, *dfrA17*, *sul2*, *gyrA* (p.S83L), *gyrA* (p.D87N), *parC* (p.S80I)	*mph(A)*, *aadA5*, *aph(3*″*)-Ib*, *aph(6)-Id*
Ec_SM78-1	AMP, TET, TMP, SMX	*bla*_TEM-1A_, *tet(B)*, *dfrA1*, *sul3*	*aadA1*
Ec_SM79-1	AMP, TET, SMX, NAL	*bla*_TEM-1B_, *tet(B)*, *sul3*, *gyrA* (p.S83A)	*mef(B)*, *aph(3*″*)-Ib*, *aph(6)-Id*
Ec_SM114-1	AMP, TET, SMX	*bla*_TEM-1B_, *tet(A)*, *sul1*	*aadA12*
Ec_SM141-1	AMP, TET, CHL, TMP, SMX	*bla*_TEM-1B_, *tet(A)*, *cmlA1*, *dfrA12*, *sul3*	*mef(B)*, *aadA1*, *aadA2*
Ec_SM142-1	AMP, TET, CHL, TMP, SMX, CIP	*bla*_TEM-1B_, *tet(A)*, *cmlA1*, *dfrA12*, *sul3*, *qnrS1*	No
Ec_SM145-1	AMP, AZM, TMP, SMX, NAL	*bla*_TEM-1B_, *mph(G)*, *dfrA5*, *sul2*, *gyrA* (p.S83L)	*aph(3*″*)-Ib*, *aph(6)-Id*
Ec_SM146-1	AMP, AZM, TMP, SMX, NAL	*bla*_TEM-1B_, *mph(G)*, *dfrA5*, *sul2*, *gyrA* (p.S83L)	*aph(3*″*)-Ib*, *aph(6)-Id*
Ec_SM147-1	AMP, TET, TMP	*bla*_TEM-1A_, *tet(B)*, *dfrA1*, *dfrA5*	*aadA1*
Ec_SM148-1	AMP, AZM, TMP, SMX, NAL	*bla*_TEM-1B_, *mph(G)*, *dfrA5*, *sul2*, *gyrA* (p.S83L)	*aph(3*″*)-Ib*, *aph(6)-Id*
Ec_SM150-1	AMP, FEP, COX, CRO, TMP, SMX	*bla*_CTX-M-1_, *dfrA17*, *sul2*	*aadA5*
Ec_SM152-1	AMP, TMP, SMX	*bla*_TEM-1B_, *dfrA5*, *sul2*	*aph(3*″*)-Ib*, *aph(6)-Id*
Ec_SM153-1	AMP, TET, TMP, SMX	*bla*_TEM-1A_, *tet(A)*, *dfrA8*, *sul2*	*aph(6)-Id*
Ec_SM154-3	AMP, TET, TMP, SMX	*bla*_TEM-1B_, *tet(A)*, *dfrA14*, *sul2*	*aph(6)-Id*
Ec_SM164-1	AMP, TET, CHL	*bla*_TEM-1B_, *tet(A)*, *floR*	*qnrS1*
Ec_SM164-3	AMP, TET, SMX	*bla*_TEM-1B_, *tet(B)*, *sul3*	*aph(6)-Id*
Ec_SM165-2	CHL, SMX, CIP, NAL	*floR*, *sul2*, *qnrS1*, *gyrA* (p.S83L)	No
Ec_SM166-3	CHL, SMX	*floR*, *sul2*	*qnrS1*
Ec_SM196-1	AMP, TET, CHL, SMX	*bla*_TEM-1C_, *tet(A)*, *floR*, *sul2*	*aph(3*″*)-Ib*
Ec_SM206-1	TMP, SMX, NAL	*dfrA1*, *sul3*, *gyrA* (p.S83L)	*aadA1*, *aph(3*″*)-Ib*, *aph(6)-Id*
**Isolate ID**	**Phenotypic profile**	**Resistance genes and point mutations related to phenotype profile**	**Other resistance genes**
Ec_CC8-1	SMX, CIP, NAL	*sul3*, *gyrA* (p.S83L), *gyrA* (p.D87N), *parC* (p.S80I)	No
Ec_CC9-5	AMC, AMP, TET, CHL, TMP, SMX	*bla*_TEM-1B_, *tet(A)*, *cmlA1*, *dfrA8*, *sul3*	*qnrS1*, *aadA1*, *aph(6)-Id*
Ec_CC10-1	AMC, AMP, TET, CHL, TMP, SMX	*bla*_TEM-1B_, *tet(A)*, *dfrA1*, *sul2*	*mph(B)*, *aadA1*, *aph(3*″*)-Ib*, *aph(6)-Id*
Ec_CC13-4	AMP, TET, SMX	*bla*_TEM-1B_, *tet(A)*, *sul3*	No
Ec_CC15-4	AMC, AMP, TET	*bla*_TEM-1C_, *tet(A)*	No
Ec_CC22-3	AMP, TET, SMX	*bla*_TEM-1B_, *tet(A)*, *sul1*	*aadA12*
Ec_CC24-1	TET, CHL, SMX, NAL	*tet(A)*, *cmlA1*, *sul3*, *gyrA* (p.S83L)	*dfrA12*, *aadA2*
Ec_CC27-4	AMP, TET, SMX	*bla*_TEM-1B_, *tet(A)*, *sul1*	No
Ec_CC28-2	AMP, TET, TMP, SMX	*bla*_TEM-1B_, *tet(A)*, *dfrA1*, *sul1*	*aadA1*
Ec_CC34-1	AMP, TET, TMP, SMX, CIP, NAL	*bla*_TEM-1B_, *tet(A)*, *dfrA14*, *sul2*, *gyrA* (p.S83L), *gyrA* (p.D87N), *parC* (p.S80I)	*aph(6)-Id*
Ec_CC44-5	TET, TMP, SMX	*tet(A)*, *dfrA14*, *sul2*	*qnrS1*, *aph(3*″*)-Ib*, *aph(6)-Id*
Ec_CC46-1	AMP, TET, SMX	*bla*_TEM-1B_, *tet(A)*, *sul2*	*aph(3*″*)-Ib*, *aph(6)-Id*
Ec_CC47-2	AMP, TET, CHL, TMP, SMX, NAL	*bla*_TEM-1C_, *tet(A)*, *cmlA1*, *dfrA12*, *sul3*, *gyrA* (p.S83L), *parE* (p.I529L)	*aadA1*, *aadA2*
Ec_CC48-2	AMP, TET, CHL, TMP, SMX	*bla*_TEM-1A_, *tet(A)*, *cmlA1*, *dfrA1*, *sul2*, *sul3*	*aph(6)-Id*
***Salmonella* spp.**			
**Isolate ID**	**Phenotypic profile**	**Resistance genes and point mutations related to phenotype profile**	**Other resistance genes**
Se_CC10	PEF, NAL	*qnrB19*, *qnrB82*	*aac(6*′*)-Iaa*
Se_CC26	AMP, TET, SMX	*bla*_TEM-1B_, *tet(B)*, *sul2*	*aac(6*′*)-Iaa*, *aph(3*″*)-Ib*, *aph(6)-Id*
Se_CC47	AMP, CHL, TMP, SMX	*bla*_TEM-1B_, *cmlA1*, *dfrA12*, *sul3*	*aac(6*′*)-Iaa*, *aadA1*, *aadA2*

SM, isolates from faeces samples; CC, isolates from raw pork meat samples; AMP, ampicillin; AMC, amoxicillin–clavulanic acid; FEP, cefepime; COX, cefotaxime; CRO, ceftriaxone; TET, tetracycline; CHL, chloramphenicol; AZM azithromycin; TMP, trimethoprim; SMX, sulfamethoxazole; CIP, ciprofloxacin; NAL, nalidixic acid.

**Table 4:** Criteria for the interpretation of the *E. coli* and *Salmonella* spp. microbiological quality results, according to the defined parameters.

Microbiological Quality Parameters	Interpretation		
	Satisfactory	Borderline	Unsatisfactory
*E. coli* count (cfu/g)	≤500	>500 and ≤5000	>5000
Presence of *Salmonella* spp. (in 25 g)	Not detected	N/A	Detected

cfu/g, colony-forming units *per* gram; N/A, not applicable.

## Data Availability

All supporting data and protocols have been provided within the article or through the Supplementary Data files. The Supplementary Material is available with the online version of this article.

## Supplementary Materials

The following supporting information can be downloaded at: https://www.mdpi.com/article/10.3390/antibiotics13100957/s1. Supplementary Table S1: Epidemiological, phenotypic, and genotypic data of the 61 *E. coli* and the 3 *Salmonella* isolates subjected to WGS; Supplementary Table S2: Virulence factors of *Salmonella* isolates obtained from the Virulence Factor Database (VFDB) server; Supplementary Table S3: Antimicrobial resistance pattern of the 139 *E. coli* isolates recovered from pork meat and from pig faeces samples; Supplementary Table S4: Primers and protocol conditions used in the PCR assays.
